# Putative Microsatellite DNA Marker-Based Wheat Genomic Resource for Varietal Improvement and Management

**DOI:** 10.3389/fpls.2017.02009

**Published:** 2017-11-28

**Authors:** Sarika Jaiswal, Sonia Sheoran, Vasu Arora, Ulavappa B. Angadi, Mir A. Iquebal, Nishu Raghav, Bharti Aneja, Deepender Kumar, Rajender Singh, Pradeep Sharma, G. P. Singh, Anil Rai, Ratan Tiwari, Dinesh Kumar

**Affiliations:** ^1^Centre for Agricultural Bioinformatics, ICAR-Indian Agricultural Statistics Research Institute, New Delhi, India; ^2^ICAR-Indian Institute of Wheat and Barley Research, Karnal, India

**Keywords:** DUS, EDV, e-PCR, molecular marker, seed purity, SSR, *Triticum aestivum*

## Abstract

Wheat fulfills 20% of global caloric requirement. World needs 60% more wheat for 9 billion population by 2050 but climate change with increasing temperature is projected to affect wheat productivity adversely. Trait improvement and management of wheat germplasm requires genomic resource. Simple Sequence Repeats (SSRs) being highly polymorphic and ubiquitously distributed in the genome, can be a marker of choice but there is no structured marker database with options to generate primer pairs for genotyping on desired chromosome/physical location. Previously associated markers with different wheat trait are also not available in any database. Limitations of *in vitro* SSR discovery can be overcome by genome-wide *in silico* mining of SSR. *Triticum aestivum* SSR database (*TaSSRDb*) is an integrated online database with three-tier architecture, developed using PHP and MySQL and accessible at http://webtom.cabgrid.res.in/wheatssr/. For genotyping, Primer3 standalone code computes primers on user request. Chromosome-wise SSR calling for all the three sub genomes along with choice of motif types is provided in addition to the primer generation for desired marker. We report here a database of highest number of SSRs (476,169) from complex, hexaploid wheat genome (~17 GB) along with previously reported 268 SSR markers associated with 11 traits. Highest (116.93 SSRs/Mb) and lowest (74.57 SSRs/Mb) SSR densities were found on 2D and 3A chromosome, respectively. To obtain homozygous locus, e-PCR was done. Such 30 loci were randomly selected for PCR validation in panel of 18 wheat Advance Varietal Trial (AVT) lines. *TaSSRDb* can be a valuable genomic resource tool for linkage mapping, gene/QTL (Quantitative trait locus) discovery, diversity analysis, traceability and variety identification. Varietal specific profiling and differentiation can supplement DUS (Distinctiveness, Uniformity, and Stability) testing, EDV (Essentially Derived Variety)/IV (Initial Variety) disputes, seed purity and hybrid wheat testing. All these are required in germplasm management as well as also in the endeavor of wheat productivity.

## Introduction

Wheat is a widely grown food grain crop of the world catering 20% of caloric need (FAO, 2016). By 2050, its global demand is expected to rise by 60% (for 9 billion population), while climate change is anticipated to adversely affect the production by 29% (Muthamilarasan et al., 2014). By 2080, global temperature is likely to increase by 4.5 degree Celsius affecting by 6% decline in productivity per degree Celsius (Asseng et al., 2015). To ensure food security, computational genomics can be integrated with wheat breeding for better wheat production along with quality and disease resistance by developing genomic resource. Such resource can be used for varietal improvement and management of germplasm.

Initially available wheat genome (2n = 6x = 42) is huge (~17 GB), complex (AABBDD, 3 homoeologous subgenomes), with high repeat content (80%) which was having more than 124 thousand genes (Consortium, 2014). As per latest pangenome-based assembly, wheat genome has 140,500 ± 102 genes, with a core genome of 81,070 ± 1,631 genes. This latest assembly has an average of 128,656 genes across 18 wheat cultivars, which has improved the reference of Chinese spring (Montenegro et al., 2017). Available *de novo* wheat genome assembly of International Wheat Genome Sequencing Consortium (Consortium, 2014) can be used as an invaluable resource to mine putative Simple sequence repeats (SSR) markers which are ubiquitously distributed over entire genome. SSRs are preferred markers for genetic diversity, linkage mapping, association studies, and marker assisted selection due to its high reproducibility, multi-allelic nature, co-dominant inheritance, robust amplification, amenability of automated/throughput genotyping with multiplexing (Li et al., 2004). All these attributes can accelerate the molecular breeding program. Genes associated with various traits like productivity, quality, biotic and abiotic stress tolerance can be used in breeding program using their flanking region SSR markers having diagnostic allele i.e., size difference in the parental lines (Twyford and Ennos, 2012).

Simple sequence repeats (SSR) located closer to the targeted genes controlling trait can be transferred in wheat variety improvement program (Leonova et al., 2011). Limited SSRs have been used in GWAS studies for 14 traits using single locus single trait (SLST), multi locus mixed model (MLMM), and multi-trait mixed model (MTMM) approach, for discovery of few QTL in wheat (Jaiswal et al., 2016). Hence, bulk mining of SSRs from each and every chromosome is required, which can be used in more QTL (Quantitative trait locus) and gene discovery by construction of linkage map with higher marker density. SSR markers can be used in varietal differentiation and traceability of the wheat produce and products (Fujita et al., 2009).

As per International Union for the Protection of New Varieties of Plants (UPOV) regulations, a “new” plant variety has a pre-requisite of being novel, Distinct Uniform and Stable (DUS) in order to enable a plant breeder to have ownership right as Plant Breeder Rights (PBR) (Yim et al., 2009). After WTO-TRIPS (World Trade Organization- Trade-Related aspects of the Intellectual Property Rights) agreement, plant variety has a global status of intellectual property. It has been implemented where DUS features, *viz*., distinctiveness, uniformity and stability are recorded and such data are used in assigning variety status as well as resolving varietal disputes (Kwon et al., 2005).

Molecular markers can not only supplement this testing (by variety specific “allelic” distinctiveness) which improves accuracy further, but sometime it can play critical role in resolving legal dispute also. Varietal status can be checked in shortest span of time rather than going for two generation trial having uncertainty of environmental fluctuations. Besides this, the other two remaining DUS components *viz*., uniformity and stability of variety in question can also be checked by horizontal and vertical distribution profile of alleles, respectively (Kwon et al., 2005). All such approaches require robust set of polymorphic SSR markers from each and every chromosome having/making unique profile of “variety signature” with respect to the other existing/disputed varieties. In many country, such SSR profiling is a pre-requisite in statutory “Germplasm Bank” as an integral part of germplasm identity and protection. Globally there are large number of varieties and such varietal differentiation also requires whole genome scanning of SSRs in order to get polymorphic markers. Molecular markers are also required for wheat seed purity testing and differentiation of hybrids (Wang et al., 2014a).

In few countries, SSR markers have been used successfully for variety differentiation. For example, >500 European wheat varieties have been differentiated by 19 SSRs (Röder et al., 2002). Limited success has also been reported to delineate group of wheat varieties/advanced variety trial (AVT) lines using few set of markers (Malik et al., 2013). Though varietal differentiation requires less number of SSRs (2–3 per chromosome) but essentially derived variety (EDV)/initial variety (IV) variety status differentiation requires much more number of markers covering each and every segment of chromosome. Limited success in differentiation of such EDVs has been reported in durum wheat (*Triticum durum*) (Noli et al., 2012). More such studies are warranted in hexaploid wheat (*Triticum aestivum*). Since phenotypic data alone cannot easily differentiate this, empirical molecular approach is the pragmatic solution. Thus, more makers are required for successful varietal differentiation, including EDVs in wheat germplasm management and improvement.

Earlier *in silico* studies on wheat whole genome-based microsatellites mining were confined to relative abundance, distribution and genomic localization using draft genome of Chinese spring wheat (Han et al., 2015; Deng et al., 2016). Though these studies have reported 364 thousand putative SSR markers, but there is no marker database having global accessibility with ready to use markers from desirable chromosome at desirable location. Even in a mega database of plant genomes (PMDbase) having 110 species has information on wheat SSR but has several major limitation for its versatile use (Yu et al., 2017). This database lacks chromosome-wise wheat genome data to obtain putative microsatellite markers at desired location/loci. It further lacks in complete automation of microsatellite mining and primer designing thus requires manual intervention in multiple steps. Besides these, it drastically compromises due to its incapability to handle more than 30 Kb query sequence size. This limitation is a great challenge for large and complex genome like wheat. Since wheat genome assembly is annotated, thus closely linked SSR markers along with their respective physical location can be used as an atlas to select appropriate choice of markers. Though *in vitro* discovered limited SSR markers have been used in wheat breeding program in last two decades, but there is no compilation of these markers in readily available form to global community.

The present work aims at genome-wide SSR mining in wheat, development of a comprehensive database having catalog of chromosome-wise SSR of all the three sub-genomes (A,B,D) along with the option of designing primers for genotyping. It also aims at compilation of all previously reported SSR markers of association studies with trait-wise retrieval option.

## Materials and methods

### Data source

The whole draft genome sequence of *T. aestivum* (cv. Chinese spring -Release 30) was retrieved in FASTA format from *Ensemblplants* (ftp://ftp.ensemblgenomes.org/pub/release-30/plants/fasta/triticum_aestivum/dna/). A total of 5011936884 sequences were downloaded and analyzed. For SSR mining, sequences were taken chromosome-wise and using PERL script, mining of microsatellite markers using MIcroSAtellite identification (MISA) tool with default parameter settings was done (Thiel et al., 2003). The primer modeling software, Primer3 (Untergasser et al., 2012) was used to identify primer pairs, taking the flanking sequences. The intermediate perl scripts of p3_in.pl and p3_out.pl were used to create Primer3 files for information retrieval. These scripts were downloaded from MISA (http://pgrc.ipk-gatersleben.de/misa/) and Primer3 (https://sourceforge.net/projects/primer3/). The default parameters for designing primers were 18–27 bp length, 57–63°C melting temperature, GC content 30–70% and product size range 100–280 bp. Further, the Primer3 core executable was integrated at the backend for primer generation of the selected markers. Availability of primer designing options further enhances the utility of database by providing the facility to generate the primers of targeted region on desired chromosome coupled with GC content, number of base pairs and copy numbers.

### Database architecture

*Triticum aestivum* SSR database (*TaSSRDb*) is an integrated online relational database with three-tier architecture having a client tier, middle tier and database tier. This user-friendly interface for the database has been developed using PHP (Hypertext preprocessor) which is an open-source server-side scripting language (Royappa and Jackson, 2000). For primer designing, primer3 standalone code computes primers on user request. Supplementary Figure 1 shows the architecture of *TaSSRDb*.

### *In silico* selection of homozygous loci and designing of primers for SSR genotyping

For wet-lab validation of SSR, locus having homozygous (similar size) alleles in reference genome assembly were searched using ePCR of the respective designed primers. While designing primer, locus covered were with >8 repeat units of simple and di-nucleotide repeat with flanking regions of 1,000 bp on each side. Care was taken to select larger and simple repeats which have higher mutation rate thus they are more polymorphic (Oliveira et al., 2006). Initially, all markers along with primers were mined from genome A. Subsequently, all these primers were put in e-PCR over the sub-genomes B and D. Locus having similar product size were filtered. All such monomorphic (identical) alleles of seven chromosomes were tabulated (Supplementary Table 1). To carry out these *in silico* steps, scripting was done in perl for string matching. In order to validate these in lab, we randomly selected 30 such loci having identical size assuming as homozygous. Primer pairs were designed for each locus.

### *In vitro* assay of SSR genotyping for validation

Samples of young leaves of 15 days old seedlings from a panel of 19 AVTs lines of year 2016–2017 were used for DNA extraction. This was done by modified CTAB method (Saghai-Maroof et al., 1984). DNA amplification was carried out in a 96 well thermocycler (Bio-Rad S1000 thermal cycler) with each reaction mixture containing 50–100 ng template DNA, 250 nM of each primer, 200 μM of each deoxynucleotide, 1.5 mM MgCl2, 1x PCR buffer and 1 U of Taq DNA polymerase. PCR was done with initial denaturation at 94°C for 3 min, followed by 45 cycles of 94°C for 1 min, 60°C for 1 min, 72°C for 2 min with a final extension step of 10 min at 72°C. Microsatellite fragments were electrophoresed in 3% (w/v) agarose gels (Himedia, USA) stained with ethidium bromide (EtBr) at 5 V/cm in 1x TBE (Tris-Borate-EDTA) buffer and visualized in Gel doc (Alpha Image EP, Alpha Innotech) system using 100 bp ladder (BioLabsinc) as standard.

## Results and discussion

From the whole draft genome sequence of *T. aestivum* (cv. Chinese spring), genome-wide SSR markers were mined. A total of 476,169 SSR markers were obtained, of which 433,362 were simple [having only one type of tandem repeats, for example (CA)15] and 42,807 were compound [having more than one type of tandem repeat, for example, (CA)8(CG)12] markers. Table 1 shows the chromosome-wise distribution of SSR markers in all the three sub-genomes. In literature, many organisms have shown non-random distribution of SSRs across the genome (Morgante et al., 2002). Microsatellite distribution showed abundance on B genome chromosomes followed by A and D genome chromosomes. Chromosome-wise results showed that chromosome 3 has the highest number of SSR markers (106,606) followed by chromosomes 2, 7, 4, 1, 5 while chromosome 6 exhibits the fewest number of SSR markers (53,440) in all homologous group. Similarly, at individual level, chromosome 3B showed highest number (80,527) of SSR markers and chromosome 4D (shortest chromosome) reported least (11,400) markers. Results also revealed that highest frequency of microsatellites was on 3B (80,527) chromosome (largest chromosome) of Chinese spring wheat followed by 2B (32,757) chromosome. These results are in congruence with the previous report of Han et al. (2015) but another study reported highest frequency of microsatellites on 2B chromosome followed by 3B in Chinese spring wheat (Deng et al., 2016). In our study, density on chromosome group 3 was found highest (98.36 SSR/Mb) and lowest (89.56) was observed on chromosome group 1 with an average density of 95.01 SSR/Mb over the genome. Individual chromosome-wise highest (116.93 markers/Mb) and lowest (74.57 markers/Mb) SSR densities were found on 2D and 3A chromosome, respectively. SSR density in wheat (*T. aestivum* L.) was reported to be 29.73 per Mb (Han et al., 2015). While previous studies in wheat and its wild relatives, namely, *T. aestivum, Triticum turgidum* (Strongfield), *Triticum turgidum* (Langdon), *Triticum urartu*, and *Aegilops tauschii*, the SSRs densities were 105.03, 144.72, 150.53, 97.85, and 101.82 SSRs/Mb, respectively (Deng et al., 2016). This variation in SSR density of wheat genome is due to different reference assembly of wheat genome having variation in length and genome finishing. In previous studies, SSR density reported in other crops, such as 52.9 SSRs/Mbp in castor bean (Tan et al., 2014), 120 SSRs/Mbp for maize (Huo et al., 2008), 370.5 SSRs/Mbp in *Arabidopsis* (Cavagnaro et al., 2010) and 111 SSR/Mbp in watermelon (Zhu H. et al., 2016). Similarly, number of repeats for each chromosome was calculated. The relative abundance in terms of percentage of dinucleotide repeats was highest while hexanucleotide were observed lowest, representing 36.10 and 0.15% of the total genome, respectively (Table 1).

**Table 1:** Chromosome-wise distribution of SSR markers in A, B, and D genomes.

**Length(bp)**	**Chr**	**mono**	**Di**	**Tri**	**Tetra**	**Penta**	**Hexa**	**Compound**	**Total**
248437066	1A	6,734	8,445	4,386	366	63	30	1,631	21,655
254944609	2A	6,981	8,923	4,472	434	70	44	1,736	22,660
185052605	3A	3,392	6,127	2,835	242	35	26	1,142	13,799
215863839	4A	7,318	7,581	4,011	347	54	37	1,525	20,873
148252673	5A	3,436	5,316	2,307	247	40	30	962	12,338
207910053	6A	6,432	7,580	3,881	317	42	30	1,492	19,774
182007620	7A	6,035	6,930	2,978	353	50	38	1,761	18,145
295113561	1B	8,696	9,980	5,250	396	49	26	2,503	26,900
345274828	2B	11,766	11,099	6,605	476	56	49	2,706	32,757
774434471	3B	30,840	25,872	14,011	1,004	142	103	8,555	80,527
317344599	4B	9,707	10,727	5,644	437	53	43	2,956	29,567
273623383	5B	9,682	8,638	5,522	407	64	38	2,149	26,500
204046169	6B	5,160	7,157	3,325	225	30	25	1,624	17,546
251517909	7B	8,146	9,050	4,008	343	47	34	2,801	24,429
135633378	1D	4,394	4,367	2,393	216	27	17	862	12,276
150381694	2D	6,995	5,659	2,890	236	30	22	1,752	17,584
124322642	3D	4,838	4,379	1,676	194	14	16	1,163	12,280
121193423	4D	3,964	3,944	2,279	162	17	8	1,026	11,400
161539237	5D	6,519	5,497	2,992	331	35	34	1,227	16,635
177000238	6D	5,220	6,164	3,149	256	27	26	1,278	16,120
238042887	7D	7,475	8,455	4,092	357	39	30	1,956	22,404
5011936884		163,730	171,890	88,706	7,346	984	706	42,807	476,169

We obtained the SSR density 95 per Mb. Wheat genome being a complex, polyploid having 80–90% repeat sequence which are excluded in the present draft assembly. Hence, SSR density of present assembly of selected regions may not hold true for the overall density of the entire genome.

Segregation distortion phenomena adversely affects linkage map construction and QTL identification. High density SSR consensus map can resolve such issue (Li et al., 2015). Such high density linkage map of wheat has been successfully used to discover QTL controling grain shape and size (Wu et al., 2015). Bulk SSRs can be used in linkage map even in single intraspecific population of common wheat (Torada et al., 2006). Our 476,169 SSRs can be used to construct densest genetic map of wheat like cotton which is an indispensable genomic resource (Khan et al., 2016).

In the present study, mono-nucleotide repeats (MNRs) and di-nucleotide repeats (DNRs) mostly contributed to the proportion of SSRs and a very small part was contributed by penta-nucleotide repeats (PNRs), and hexa-nucleotide repeats (HNRs). In terms of the distribution of different motifs, among DNRs GA had the higher occurrence (8,012) followed by CT (7,493) and TC (7,258). In TNRs category, most frequent motif type found was GAA (3,606) followed by CTT (3,302), GAG (2,294), AAG (1,908) and CCT (1,904). For tetra-nucleotide repeats (TeNRs), CATG (291), TGCA (254), TTTA (170), AAAT (157), and TTTC (99) were observed more frequently while in HNRs, TATAGA/TCTATA showed highest frequency. AT-rich repeat patterns were observed among PNRs and HNRs (Table 2). Hence, dinucleotide repeats showed the highest among all the repeats (34.30 SSR/Mb). Similar finding of higher frequency of DNRs in wheat (*T. aestivum*) and its progenitors (*T. uratu* and *A. tauschii*) are also reported (Deng et al., 2016). These results are in congruence with few previous other plant reports such as in *Citrullus lanatus* (Zhu H. et al., 2016) but dissimilar with the few other species (*Glycine max, Arabidopsis thaliana, Oryza sativa*, and *Sorghum bicolour*) in which trinucleotide repeats were reported highest and in cucumber, *Medicago truncatula, Populus trichocarpa*, and *Vitis vinifera*, where tetra-nucleotide repeats were observed highest (Zhu H. et al., 2016). It is reported that TNRs and TeNRs were found abundant in monocotyledonous species while MNRs and DNRs were observed highest in dicotyledonous species (Shi et al., 2013). The polymorphism level in SSR loci is positively correlated with the increasing number of repeat units in microsatellites (Tan et al., 2014).

**Table 2:** List of abundant repeats.

	**mono**		**Di**		**Tri**		**Tetra**		**Penta**		**Hexa**	
	**Repeat**	**Absolute number**	**Repeat**	**Absolute number**	**Repeat**	**Absolute number**	**Repeat**	**Absolute number**	**Repeat**	**Absolute number**	**Repeat**	**Absolute number**
Repeat 1	A(10)	32,883	GA(6)	8,012	GAA(5)	3,606	CATG(5)	291	AAAAT(5)	23	TATAGA(5)	27
Repeat 2	T(10)	32,790	CT(6)	7,493	CTT(5)	3,302	TGCA(5)	254	AAAAG(5)	21	TATAGA(6)	16
Repeat3	G(10)	16,513	TC(6)	7,258	GAG(5)	2,294	TTTA(5)	170	TTTTC(5)	21	TATCTA(5)	16
Repeat4	C(10)	16,023	AT(6)	6,970	AAG(5)	1,908	AAAT(5)	157	CTTTT(5)	15	ATCTAT(5)	14
Repeat5	G(11)	10,522	TA(6)	6,927	CCT(5)	1,904	TTTC(5)	99	AAAGA(5)	14	TCTATA(5)	13

**Figure 1:**
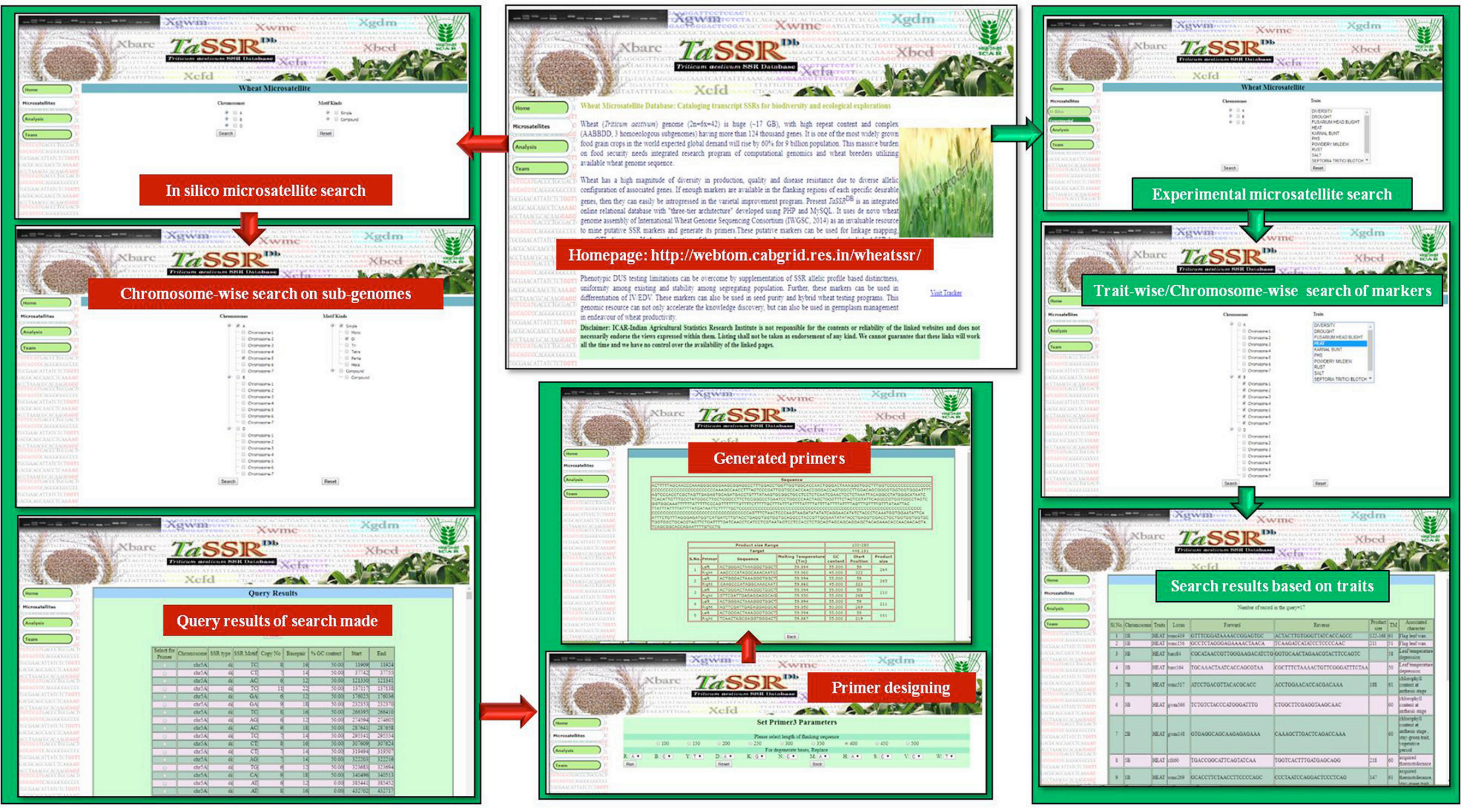
The web interface of *TaSSRDb* showing microsatellite search, its result, generated primers and trait linked SSR search.

*TaSSRDb* is mainly equipped with four tabs (Home, Microsatellites, Analysis and Team). Users are provided with two options, *viz., in silico* and experimental under the “Microsatellite” tab. *In silico* is used for mining of simple and compound chromosome specific SSRs markers, which includes the information on the sequence, type, motif, copy number, base pair, percentage GC content and physical position (start and end) of microsatellite repeat. Additionally, for designing primers of desired marker, Primer3 integrated at the backend provides primers of desirable product size, annealing temperature and GC content. The “Experimental” markers displays the pre-existing markers in literature, which are cataloged based on the chromosome-wise distribution on all the three wheat sub-genomes, with their loci and the traits with which these are associated. The different traits of association are drought, *Fusarium* head blight (FHB), heat, Karnal bunt (KB), pre-harvest sprouting (PHS), powdery mildew, rust, salinity, *Septoria tritici* blotch, and spot blotch. Figure 1 shows the detailed workflow for the search of markers. *TaSSRDb* has >470 thousand SSR loci where primer can be designed for genotyping for association studies. Associated SSR loci can be used in trait improvement by marker assisted gene introgression program in varietal development. Such use of SSR primers in 10 different wheat traits are populated in our database. So far atleast >10 wheat traits are reported where similar use of SSR has been very successful. These wheat traits are viz., FHB (Wiśniewska et al., 2016; Zhu Z. et al., 2016), heat (Mondal et al., 2015; Awlachew et al., 2016), KB (Kumar et al., 2007; Kaur et al., 2016), PHS (Shorinola et al., 2016), powdery mildew (Miranda et al., 2006), rust (Roder et al., 1998); salinity (Shahzad et al., 2012; Ghaedrahmati et al., 2014), Septoria tritici blotch (Roder et al., 1998), Spot blotch (Kumar et al., 2015; Singh et al., 2016), and drought (Peleg et al., 2009; Pinto et al., 2010).

### SSR validation

Using e-PCR, a total of 2013 identical alleles were obtained in wheat genome having an average of 96 SSRs per chromosome in each sub-genome. Such monomorphic SSR markers are most valuable genomic resource of the variety from which it has been mined. Such loci having size difference in a set of parental lines can directly be used in molecular breeding program (Han et al., 2015).

**Figure 2:**
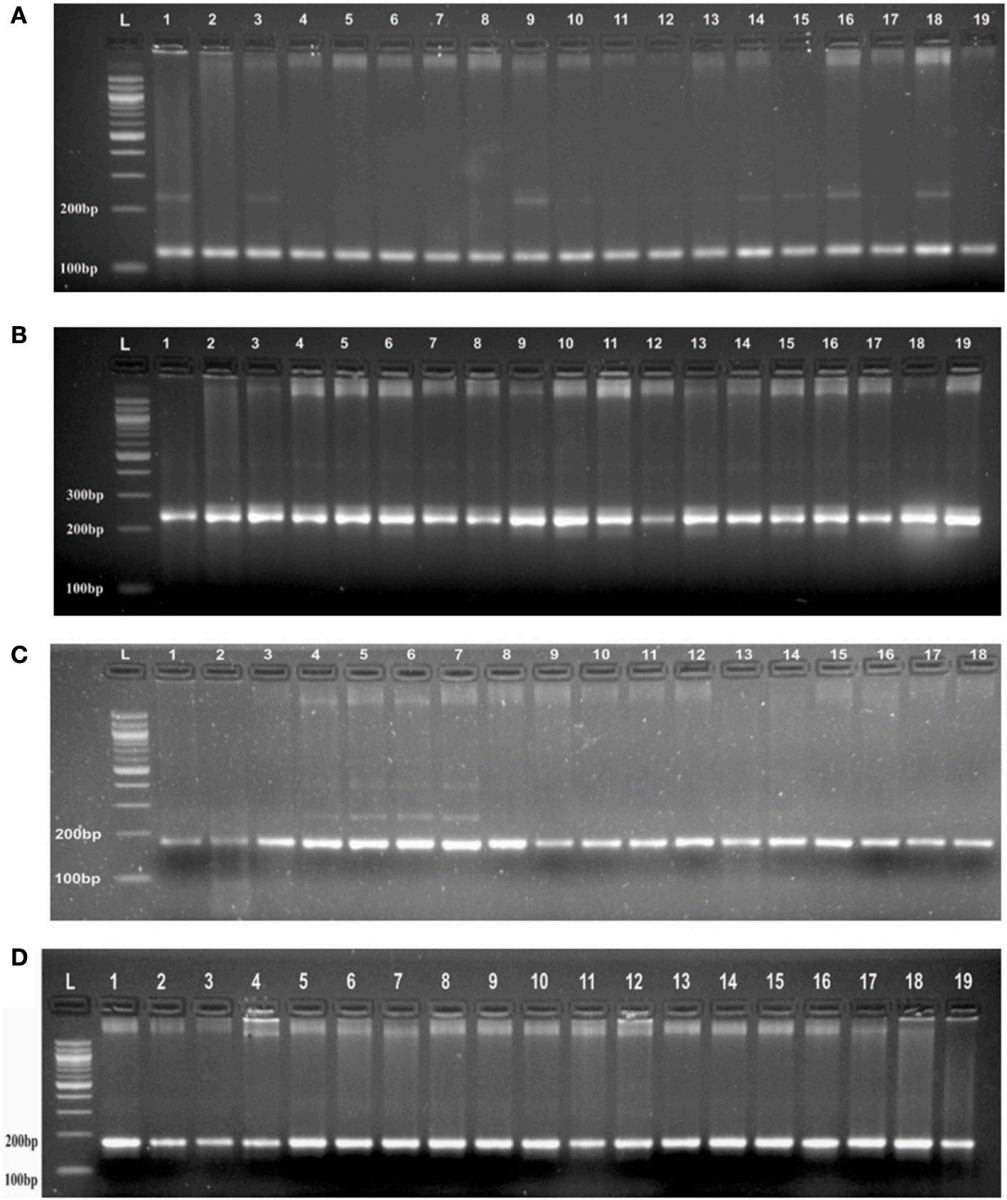
**(A)** PCR profile of SSR marker (GA)_8_ from Chromosome 1 in AVT lines of wheat in agarose gel having resolving power (<6 basepairs cannot be detected). **(B)** PCR profile of a SSR marker (CA)_6_ from Chromosome 1 in AVT lines of wheat agarose gel having resolving power (<6 basepairs cannot be detected). **(C)** PCR profile of a SSR marker (AG)_7_ from Chromosome 3 in AVT lines of wheat agarose gel having resolving power (<6 basepairs cannot be detected). **(D)** PCR profile of a SSR marker (AC)_8_ from Chromosome 2 in AVT lines of wheat agarose gel having resolving power (<6 basepairs cannot be detected).

Genotyping of randomly selected 30 loci in a panel of 19 AVT lines gave at least three banding patterns with their respective size of PCR product in 18 AVT lines (Figure 2, Supplementary Table 2). The number of alleles observed might be less in number due to low resolving power (<6 basepairs cannot be detected) of the gel used. It is interesting to observe that though we selected monomorphic alleles having identical size, but we obtained heterozygous alleles having size difference. It is expected to vary with different varieties as each variety has a specific microsatellite profiling as signature of that variety (Röder et al., 2002). As the testing panel was AVT lines derived from different parents in varietal improvement programs, thus they are likely to have different allele size and some of them even may have heterozygous allelic configuration.

Since the draft assembly of Chinese spring wheat with more than 124 thousand genes are already available with their physical location (Consortium, 2014) thus *TaSSRDb* can be a valuable tool to search location specific putative SSR markers closely linked to targeted genes in variety improvement program. Such program requires diagnostic alleles (allele size difference) in parental population. Such trait improvement has been reported using classical breeding in wheat for salinity tolerance using locus for gene Kna1, (controlling K^+^/Na^+^) accumulation in leaves (Dubcovsky et al., 1996).

Our database is populated with such more information related to 11 wheat traits and association of 263 SSR markers. This database can be a valuable genomic resource accelerating such research as we have an atlas of SSR loci with highest density reported so far over physical map of wheat genome. Using this resource, more such association studies can be done supplementing classical breeding programs. Such introgression program requires discovery of polymorphism at SSR loci closely linked to the targeted genes in initial parental lines.

Our database can be used for diversity analysis of wheat germplasm. SSR are more efficient in diversity analysis than SNP (Singh et al., 2013). Faster mutation rate of SSR marker (average 5.5 × 10^−9^ substitutions nt^−1^ generation^−1^), with respect to SNP marker (2.4 × 10^−4^ repeats allele^−1^ generation^−1^) make them a marker of choice for recently diverged population (Chao et al., 2009).

Though SNP chip of wheat is available (35, 90, and 820 K) but for variety level differentiation and identity disputes still SSR is efficient and economical solution with equally valid relevance (Vieira et al., 2016). In some cases such varietal differentiation has been reported to increase the price tag value of wheat in the extent >2-folds in domestic and international market. Sharbati group of varieties which are well known for softness and taste (Dixon, 1974) are represented by many individual varieties like C 306, Sujata, JW3211, JW3288, HI 1531 etc., Such wheat varieties fetch better return to farmers due to higher demand/price value. Our database can be used for such wheat varietal differentiation. Besides varietal differentiation, use of SSR has been reported to detect species adulteration of soft wheat and durum wheat (Pasqualone et al., 2007). Similar use has also been reported for quantification of soft wheat adulteration in durum wheat products semolina, pasta, and bread (Sonnante et al., 2009). Such value addition and prevention of adulteration/export certification of commercially relevant other agricultural crop produce like basmati rice (Kaushik et al., 2011) are already in practice in few countries. A salt tolerant rice variety CSR10 and Taraori Basmati (HBC19) has been successfully differentiated by SSR marker in genotyping (Rana et al., 2009). Such approach has been found relevant in protecting the premium tag value of crop produce by screening the adulteration (Archak et al., 2007).

Distinctiveness, Uniformity and Stability are required in DUS testing program while assigning variety status and resolving varietal disputes. Our database can be used as a research tool to develop set of polymorphic markers having “allelic” distinctiveness by molecular profiling. Such supplementation of DUS test has already been reported in wheat (Wang et al., 2015) and in other crops like capsicum (Kwon et al., 2005), *Brassica sinensis* (Yim et al., 2009), and tomato (Cooke et al., 2003). This approach has an advantage of varietal dispute resolution in shortest span of time obviating the need of two generation trial having uncertainty of environmental fluctuations. Further, attributes of uniformity and stability of variety can also be established by such approach. It is impossible to test the stability of large number of wheat varieties with accuracy and quickness in shortest period of time unless SSR markers are used for this attribute. Such stability is assessed by ratio of homozygous SSR loci (Wang et al., 2014a).

In a cross pollinated crop like *Brassica sinensis*, variety identification has been greatly challenging. In such case, SSR has been very successfully used to assess and establish DUS feature with advantage of cost, time and other logistic loads (Yim et al., 2009). Our database can be used to get ubiquitous polymorphic SSR markers to obtain “variety signature” of wheat by global wheat community. Such wheat variety signatures are reported by Röder et al. (2002) for 500 varieties using 19 SSR markers (Röder et al., 2002). Group of Indian wheat varieties/AVT lines have been differentiated using limited set of markers (Malik et al., 2013). Such use of SSR profiling in uniformity testing to confirm DUS test of variety has already been reported in wheat (Wang et al., 2015) and tomato (Cooke et al., 2003). For similar kind of DUS feature assessment of global wheat varieties, our *TaSSRDb* can be a valuable genomic resource in wheat germplasm management.

Seed purity testing is required in seed production, market supervision and breeding research programs. SSR marker-based purity testing may have great advantage in saving time with high degree of certainty (Wang et al., 2014b). Such DNA test can enforce legal/statutory compliance in the interest of consumer, seller and variety protection and management authority.

Since morphological traits and heterosis are less suited for identification of essentially derived varieties (EDVs) in crop thus molecular markers are required to discriminate the EDV (Heckenberger et al., 2005). EDV and IV differentiation involves two different concepts, i.e., distinctness and essential derivation (Tonapi et al., 2006). Most of the wheat growing countries including India are yet to have approved standards and procedures to determine EDV status. According to UPOV Art 14(b), “Derived” means that genetic materials of the INV have been used in the creation of the later variety. Further, Article 14(5)(c) describes with examples that derivation should remain confined to one or very few characters, excluding the majority of essential characteristics of the variety. Such differentiation critically needs standardized DNA markers (AFLP, SSR, SNP etc.,) to trace each and every chromosomal segments from the IV to their derived progeny. Molecular markers can give best empirical evaluation of genetic relatedness rather than DUS phenotypic features (Heckenberger et al., 2003). For EDV/IV differentiation, DNA marker generated data can be further standardized for different species specific threshold “colors” (Heckenberger et al., 2002) by the wheat breeding community. Such analysis needs application of genetic distancing, Jaccard method, Principal Component Analysis, Principal Co-ordinate Analysis etc. on SSR data (Leigh et al., 2003).

Differentiation of EDVs has been reported by both two marker system (Heckenberger et al., 2006) as well as single marker system (Kahler et al., 2010) depending upon the marker density and degree of polymorphism. In comparison to variety differentiation, EDV/IV differentiation needs huge set of polymorphic markers covering every segment of wheat chromosome. *TaSSRDb* can be a valuable genomic resource for such marker need for global wheat community.

SSR markers can be used in program of hybrid wheat management especially to detect/monitor contamination by using homozygosity of parent lines producing hybrids. Since molecular markers are very sensitive and specific thus even lowest contamination can be detected which is not feasible otherwise by phenotypic methods. In such program, SSRs have also been used to detect cytoplasmic sterility genes/restorer genes in hybrid wheat (Sinha et al., 2013).

## Conclusion

We report world's first online web genomic resource *TaSSRDb*, (http://webtom.cabgrid.res.in/wheatssr/) of complex and hexaploid wheat genome which is having highest number (476,169) of novel putative SSR markers reported so far. We also report all existing wheat trait (11) associated markers (268 SSRs). This genomic resource can be used for linkage mapping, gene/QTL discovery, diversity analysis, traceability, variety identification, DUS testing, differentiation of EDV/IV, seed purity and hybrid testing. This genomic resource will accelerate the knowledge discovery with germplasm management. It offers user defined ready to use primer for SSR locus genotyping over desired chromosome and motif type at particular location in three sub genomes. Use and efficacy of genomic resource for genotyping has been validated by wet lab PCR after selecting 30 homozygous loci by e-PCR using panel of 18 wheat AVT lines. Trait improvement and management of wheat germplasm requires such genomic resource in the endevour of global demand gap of wheat productivity in the era of climate change and global germplasm management having sovereignty issues.

## Availability

http://webtom.cabgrid.res.in/wheatssr/

## Standard of reporting

The data used in the study are from public domain, which has been mentioned along with the source in the manuscript.

## Author contributions

DiK and RT conceived the theme of the study. VA, SJ, MI, and UA did the computational analysis and developed database. NR, BA, DeK, SS, and RT collected DNA sample and did wet lab validation. SJ, SS, MI, and DiK drafted the manuscript. RS, PS, GS, RT, and AR edited the manuscript. All co-authors read and approved the final manuscript.

### Conflict of interest statement

The authors declare that the research was conducted in the absence of any commercial or financial relationships that could be construed as a potential conflict of interest.

## Supplementary material

Supplementary Figure 1 Three-tier architecture of *TaSSRDb*.

## Abbreviations

SSR: Simple sequence repeats
PCR: Polymerase chain reaction
QTL: Quantitative trait locus
EDV: Essentially Derived Variety
IV: Initial Variety
DUS: Distinctiveness, Uniformity and Stability
GWAS: Genome, Wide Association Studies
SLST: Single locus single trait
MLMM: Multi locus mixed model
MTMM: multi, trait mixed model
UPOV: Union for the Protection of New Varieties of Plants
WTO-TRIPS: World Trade Organization, Trade Related aspects of the Intellectual Property Rights
AVT: Advanced Variety Trial
EDTA: Ethidium Bromide
MNRs: Mono Nucleotide Repeats
DNRs: Di Nucleotide Repeats
PNRs: Penta Nucleotide Repeats
HNRs: Hexa Nucleotide Repeats
TeNRs: Tetra Nucleotide Repeats.
