# Supplementary figures and images for: Putative Microsatellite DNA Marker-Based Wheat Genomic Resource for Varietal Improvement and Management
Source: Front Plant Sci. 2017 Nov 28;8:2009. doi: 10.3389/fpls.2017.02009 (PMC5712362; doi:10.3389/fpls.2017.02009)

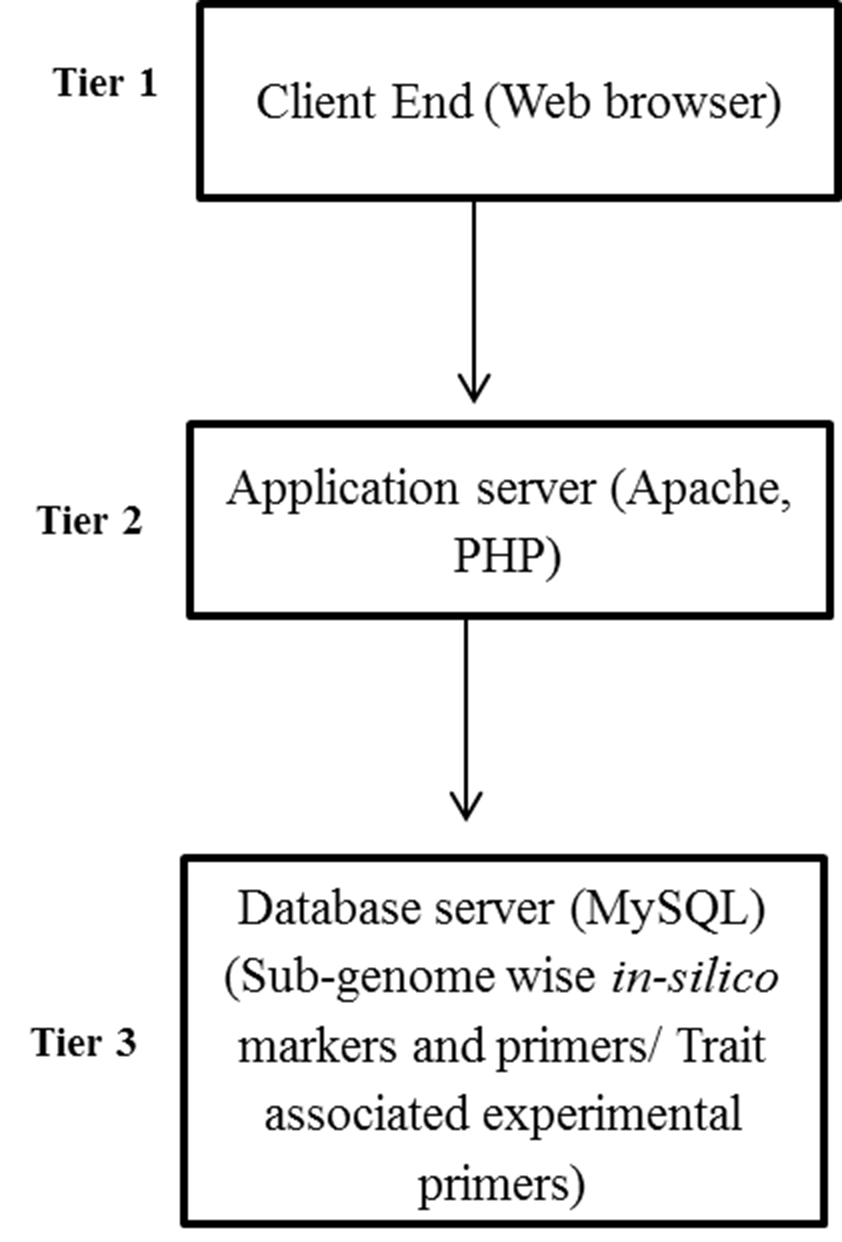

Supplement: Supplementary Figure 1 — Three-tier architecture of TaSSRDb. [file Image1.tif]
