# Supplementary material for: Putative Microsatellite DNA Marker-Based Wheat Genomic Resource for Varietal Improvement and Management
Source: Front Plant Sci. 2017 Nov 28;8:2009. doi: 10.3389/fpls.2017.02009 (PMC5712362; doi:10.3389/fpls.2017.02009)
Supplement: Supplementary file 3 [file Table2.pdf]

Supplementary Table 2. Genotyping of randomly selected 30 loci in a panel of 18 AVT lines

Chromo-some	Marker Details				Genome A	Genome B	Genome D	Product Size	
	SSR	SSR Length	Forward Primer	Reverse Primer	Start	Start	Start	Predicted*	Observed
1	(AG)24	48	GAGGGGTCGTTTGTGTCCAT	TGTCTCCTTGTGCACCACTC	177803161	21087450	22640429	288	290
1	(CA)6	12	TCCCTGCCACACACAAACTT	GAGGATCAAGTGGGTGGGTG	161185201	267125	24606396	133	140, 160
1	(CA)6	12	GACCGAGGGAATTGAACCGT	GCCAGAATTCCAGCCCTAGG	181133347	2507344	19807081	132	140, 170
1	(CA)6	12	ATAGATGCATGCTCCAGCCC	AACGTGCATGTGTGAGAGGT	86446784	29194558	23524676	228	230
1	(CA)8	16	AGCCAATGGGAGGGAACAAG	GTCCAGGTGAGTGTGATGCA	6569321	285141490	32888922	134	140, 290
1	(CT)15	30	ATCAGAGCCGCATGTACCAG	GTTGGTGCACCTAGGGTACC	36073570	196487390	44942442	213	150, 210
1	(CT)7	14	CCCTGCCACACACAAACTTG	GAGGATCAAGTGGGTGGGTG	161184917	267126	24606397	132	140
1	(GA)8	16	TGCCGGCCGTTGGATATAAA	GCCACATTTTTGCAGGCTGA	84903326	20286169	11069446	121	121, 210, 450
1	(TC)31	62	GAACAAGAGGCGTCTCCCAA	TGCCGTCATGCAAGCTTAGA	177188112	116196451	67147411	133	130
1	(TC)9	18	CAGATCCGGCTGGTATGGAC	CATGGAGCGGCTACCTTGAA	24288641	1222533	11100423	105	105
1	(TG)6	12	GCTGAAGGATCTGGAGGCAG	AGACCACATGGGAGCTGTTG	231981688	267593885	123341765	277	280
2	(AC)8	16	CTTCTCACATGCTGGCCAGA	CACAGAGGGAGCGGAAAAGT	200690303	19188094	14083469	173	180
2	(AG)6	12	GCAACTATGGTGCCTGGAGT	TCTGCCTCTACCTGAGCTGT	6310182	5663683	5520159	106	110, 120, 220
2	(AT)6	12	GGGGTGTGTCTACCGTGAAG	GTGCGTTGCAGAGGGAGTAT	156883775	35463944	18377538	225	220
2	(CT)10	20	CTACGCCACTGGTGTCAAGT	CCAACACTGTTTTGGCCCAG	39441518	184529250	93489393	106	110
2	(CT)6	12	GTTATGGCGTCCGTGAATGC	AGCGCCCCAGTGTTATCAAA	43188720	212719130	78680784	110	110, 180

2	(GA)34	68	TCCCAAACTGT TGCCATCGA	TTACCTGCCCT TTCAGCGAG	237721557	303374254	138047482	174	170
2	(GA)7	14	AGTTGACATGT CGGTCCCAC	TCTGATGGCTC TCTCCACGA	20662001	7611229	2870209	178	180
2	(GT)6	12	CCTGAACTATG CAGCCACCA	ATTGTGGTGCA CTCGGTGAT	188599651	325688110	144222019	136	140
2	(GT)7	14	GCTGGCCCAC AAAAGCTAAC	CATCTCAATCC CCTCCTGGC	218212201	28130212	17107642	144	150
3	(AG)7	14	TCACCGCATAC ACATGAGCA	TGGCAAGCAT CATGTGGTGA	67300084	19195983	5548086	175	180
3	(AG)9	18	AGCCCATGTGT GTGTGAGAG	ATGTCTCTCTC CCGCTCCTT	128702332	2916488	34930991	191	190
3	(AT)6	12	CCAATGTGGG TAACAGGCCT	TTCCCCTAACT CGCCGAGTA	119331903	367984383	44894940	116	120
3	(CA)6	12	GACGCCCACTT AAGCAGACA	GGAGGGGAAT GAATGCAGCT	84401173	39634948	4125644	118	120
3	(CT)8	16	GATGAACCGG AACAGAGCCA	GAAGATCCAA GTTGGCCCGA	14816514	15808976	93606936	211	190, 210
3	(GA)6	12	AGCCCATGTGT GTGTGAGAG	ATGTCTCTCTC CCGCTCCTT	128702162	2916488	34930991	191	190
3	(GA)7	14	AGAGCAAGGC AGGTTTCGAA	GGGTGTAGGT GCATCGTTCA	12638952	31039753	14291162	234	240
3	(GT)6	12	ATGCCGACTG GTAGTGTGTG	GCTCGTTTCCA AACCACACC	81689412	8729881	18980417	211	210
3	(TA)7	14	GAAAATGGGT TGGCATGCCA	ATTGCCCTCTG CTCACTTCC	116484804	6448255	9675763	111	110, 210, 260
3	(TG)11	22	ATGGTCGTTGG ATGAAGCCA	ACATGTGGCA AAATCTGGTG G	113892999	32927613	17518844	101	100, 200, 300
* Selected monomorphic loci from homeologous group of reference genome									
